# Multiple introductions of the dengue vector, *Aedes aegypti*, into California

**DOI:** 10.1371/journal.pntd.0005718

**Published:** 2017-08-10

**Authors:** Evlyn Pless, Andrea Gloria-Soria, Benjamin R. Evans, Vicki Kramer, Bethany G. Bolling, Walter J. Tabachnick, Jeffrey R. Powell

**Affiliations:** 1 Department of Ecology and Evolutionary Biology, Yale University, New Haven, Connecticut, United States of America; 2 California Department of Public Health, Vector-Borne Disease Section, Sacramento, California, United States of America; 3 Texas Department of State Health Services, Arbovirus-Entomology Laboratory, Austin, Texas, United States of America; 4 Florida Medical Entomology Laboratory, Department of Entomology and Nematology, University of Florida, Vero Beach, Florida, United States of America; Santa Fe Institute, UNITED STATES

## Abstract

The yellow fever mosquito *Aedes aegypti* inhabits much of the tropical and subtropical world and is a primary vector of dengue, Zika, and chikungunya viruses. Breeding populations of *A*. *aegypti* were first reported in California (CA) in 2013. Initial genetic analyses using 12 microsatellites on collections from Northern CA in 2013 indicated the South Central US region as the likely source of the introduction. We expanded genetic analyses of CA *A*. *aegypti* by: (a) examining additional Northern CA samples and including samples from Southern CA, (b) including more southern US populations for comparison, and (c) genotyping a subset of samples at 15,698 SNPs. Major results are: (1) Northern and Southern CA populations are distinct. (2) Northern populations are more genetically diverse than Southern CA populations. (3) Northern and Southern CA groups were likely founded by two independent introductions which came from the South Central US and Southwest US/northern Mexico regions respectively. (4) Our genetic data suggest that the founding events giving rise to the Northern CA and Southern CA populations likely occurred before the populations were first recognized in 2013 and 2014, respectively. (5) A Northern CA population analyzed at multiple time-points (two years apart) is genetically stable, consistent with permanent *in situ* breeding. These results expand previous work on the origin of California *A*. *aegypti* with the novel finding that this species entered California on multiple occasions, likely some years before its initial detection. This work has implications for mosquito surveillance and vector control activities not only in California but also in other regions where the distribution of this invasive mosquito is expanding.

## Author summary

Infectious diseases transmitted by *Aedes aegypti*, also known as the yellow fever mosquito, are of growing concern in tropical and subtropical regions. Dengue and Zika incidences are increasing, and no vaccines are currently available. Here we investigate the origin of California *A*. *aegypti* and find that this mosquito likely entered California on multiple occasions, at least once from the South Central US region and once from the Southwest US/northern MX region. The evidence suggests that the first invasion event likely occurred some years before its initial detection in 2013, despite California’s extensive and active surveillance program, implying that this invasive mosquito can go undetected. Understanding the invasion dynamics, gene flow, and population structure of *A*. *aegypti* can improve the monitoring of mosquitoes and prevent outbreaks of vector-borne disease.

## Introduction

Dengue, Zika, and chikungunya are severe mosquito-borne infectious diseases that are of growing concern in tropical and sub-tropical regions, and yellow fever is re-emerging in many regions [1]. The viruses causing these diseases are primarily transmitted by the mosquito vector *Aedes aegypti*. The incidence of dengue has increased 30-fold in the past 50 years [2] with an estimated 96 million new cases annually [3]. More than 2 billion people are at risk of infection by one of the four dengue serotypes [4]. Following in the footsteps of a widespread chikungunya epidemic in Asia and the New World, Zika virus has rapidly emerged around the globe. It has spread to more than 40 countries, in some cases causing serious birth defects including microcephaly [5, 6]. With no effective vaccines and limited antiviral therapeutics available for these diseases, vector control remains critically important.

The ancestor of the domestic form of *A*. *aegypti* is a zoophilic sub-species called *formosus* [7]. It is likely from sub-Saharan Africa, where it can still be found [7]. Outside of Africa, *A*. *aegypti* is a domestic mosquito that primarily bites humans, lays eggs in manmade water-containers, and can disperse over long distances using human transportation systems [8]. As such, it is a highly successful invasive species that has colonized most tropical and subtropical regions [7, 8]. *A*. *aegypti* likely migrated to the Americas in European slave ships in the fifteenth through seventeenth centuries [7, 9], and these ships probably provided an environment that helped select for traits that increased the success of the domestic form of *A*. *aegypti* [7]. The first documented epidemic of yellow fever in the New World was in the Yucatan in 1648, and yellow fever was common in Atlantic seaports from the seventeenth through nineteenth centuries, presumably fueled by the arrival of African slaves and infected sailors [8]. Today, populations of *A*. *aegypti* are distributed throughout most of the southern United States, especially below the 33-degree north latitude line [10]. Populations are sporadically found in the Mid-Atlantic States and New England, including an overwintering population in Washington D.C. [10, 11]. Locally transmitted disease from *A*. *aegypti* is not common in the United States, but there were locally transmitted cases of Zika in Miami-Dade County, Florida and Cameron County, Texas in 2016 [12]. There has been local transmission of dengue and chikungunya in Florida as recently as 2013 and 2014, respectively [13, 14].

Population genetics plays important roles in both understanding the natural history of *A*. *aegypti* and in implementing vector control. Validated genetic markers can be used to determine the source of new invasions [15, 16], detect bottlenecks potentially caused by vector control or founder effect, determine a population’s susceptibility to different classes of insecticide, infer connectivity or isolation between populations, and determine if seasonal appearance of *A*. *aegypti* is due to new introductions each year or overwintering. Mosquitoes are more likely to be present and abundant in an area where they can survive the winter, and overwintering is especially concerning in cases where the vectored viruses can also persist through the winter, either in inseminated females or through vertical transmission from mother to eggs. (There is evidence for transovarial transmission of dengue and vertical transmission of Zika in *A*. *aegypti* [17, 18].) A stable, overwintering population would be expected to be genetically stable from year to year, although this can be difficult to distinguish from a population that is re-founded each year from the same source population.

California (CA) has an extensive mosquito-monitoring program, and historically *A*. *aegypti* were only occasionally detected near airports and other ports of entry [19]. Breeding populations were first reported in 2013 from Fresno, Madera, and San Mateo. Gloria-Soria et al. concluded that the likely origin of these populations was the South Central US, particularly Houston or New Orleans [20]. Through the end of 2016, *A*. *aegypti* had been found in 96 cities and census designated places from 12 different counties in CA [21, 22].

**Fig 1:**
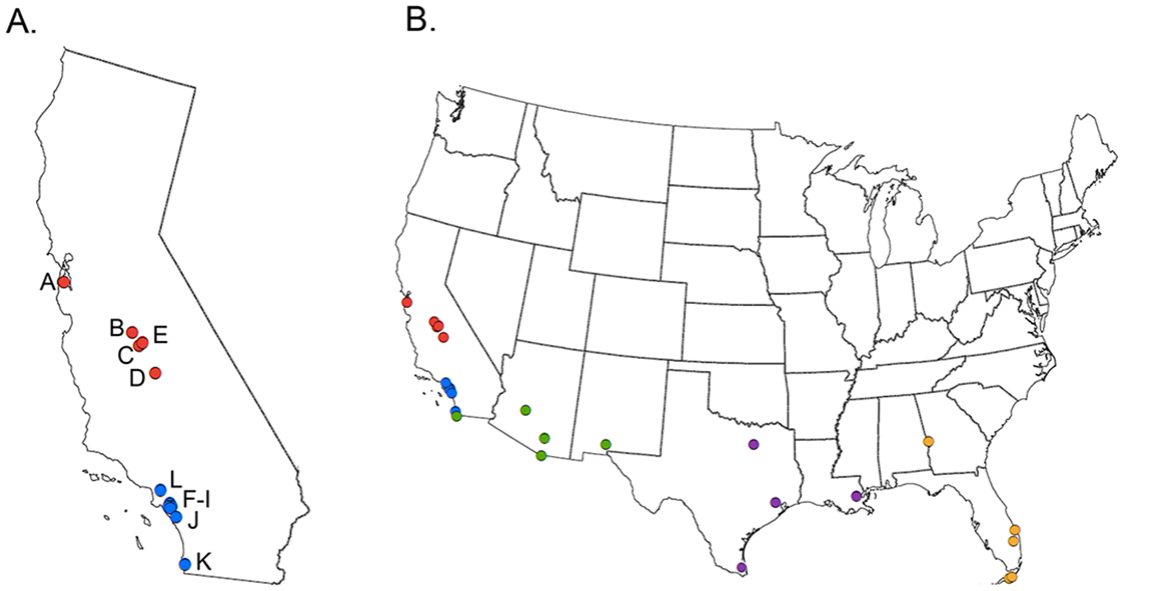
Maps of geographic locations of *Aedes aegypti* populations used in these analyses. (A) Geographic locations of the California populations. Letters refer to each city: A = San Mateo, B = Madera, C = Fresno, D = Exeter, E = Clovis, F = Anaheim, G = Orange, H = Garden Grove, I = Santa Ana, J = Mission Viejo, K = San Diego, L = Los Angeles. (B) Geographic locations of all populations included in the analyses. Colors correspond to regions: red = Southern California, blue = Northern California, green = Southwest, purple = South Central, and yellow = Southeast.

In this analysis, we have built upon our previous work by adding 13 new samples from CA, including 8 from southern CA (Fig 1A). The primary goals of the study are 1) to determine whether *A*. *aegypti* populations in CA originated from a single or from multiple introductions, 2) to characterize the genetic structure of CA *A*. *aegypti* populations, and 3) to determine if the genetic data are consistent with overwintering by *A*. *aegypti*, especially in the northern parts of CA. We found clear genetic differentiation between the Northern and Southern CA populations and found support for the hypothesis that at least two introductions of *A*. *aegypti* into CA are responsible for the current populations within the state.

## Methods

### Mosquito collections

A total of 34 samples of *A*. *aegypti* mosquitoes from 12 sites in CA and 16 sites from across the southern United States and northern Mexico were considered in analyses (Table 1). The mean sample size per collection was 39 individuals (range: 6–150). Ten of the California samples were collected between May and September of 2015, and an additional eight were collected in 2013–2014. In several cases, multiple collections were made from the same site in different years, or in different areas of the same site in a single year (as noted in Table 1). All mosquitoes from 2015 were collected as adults or eggs from traps and were shipped as adults to our laboratory for analysis. The collections made prior to 2015 are described elsewhere [15, 16, 20]. To avoid biased sampling of siblings, when ovitraps were the source of our sampling we used eggs from four or more traps from any locality with no more than six genotyped individuals per trap. Given that *Aedes aegypti* are “skip ovipositors” (normally laying one or a few eggs in multiple containers) [23], the use of multiple traps should be sufficient to minimize the sampling of siblings.

**Table 1:** *Aedes aegypti* populations included in this study.

Population/ Collection site	Year Collected	Region [Fig 1 letter]	N	Analyses	Population Abbreviation
San Mateo, CA	2013	Northern California [A]	22	SNP, ABC	SM and SM2
San Mateo, CA	2014	Northern California [A]	7		SM14
Madera, CA (1)	2013	Northern California [B]	50	SNP, ABC	CMad
Madera, CA (2)	2013	Northern California [B]	27		Mad1
Madera, CA	2015	Northern California [B]	40	SNP	MAD15
Fresno, CA	2015	Northern California [C]	34	ABC	Fres
Exeter, CA	2014	Northern California [D]	23	SNP	Exe
Clovis, CA (1)	2013	Northern California [E]	60	SNP	Clovis
Clovis, CA (2)	2013	Northern California [E]	35		Cal
Anaheim, CA (1)	2015	Southern California [F]	15	ABC	Ana_LC
Anaheim, CA (2)	2015	Southern California [F]	16	ABC	Ana
Orange, CA	2015	Southern California [G]	13	ABC	Or
Garden Grove, CA	2015	Southern California [H]	29	SNP, ABC	GG
Santa Ana, CA	2015	Southern California [I]	30	ABC	SA
Mission Viejo, CA	2015	Southern California [J]	51	SNP, ABC	MV
San Diego, CA (1)	2015	Southern California [K]	28	SNP, ABC	Cw
San Diego, CA (2)	2015	Southern California [K]	30	SNP, ABC	SY
Los Angeles, CA	2014	Southern California [L]	6	SNP	GLA
Maricopa County, AZ	2013	Southwest	53		Az
Tucson, AZ	2012	Southwest	54	ABC	TJC2
Las Cruces, NM	2015	Southwest	54	ABC	Cruc
Nogales, SON, MEX	2013	Southwest	51	ABC	Nog
Tijuana, BC, MEX	2013	Southwest	20		Tj
Houston, TX	2011	South Central	19	ABC	Houston and H
Cameron County, TX	2015	South Central	60	ABC	Cam
Dallas County, TX	2015	South Central	60	ABC	Dall
New Orleans, LA	2011	South Central	46	ABC	NO and Nola
New Orleans, LA	2012	South Central	150		N02 and D10w24
Musco, GA (1)	2011	Southeast	48		18 and 9
Musco, GA (2)	2012	Southeast	7		Musco bot.2 and GA
Rio, FL	2014	Southeast	51		FLO
Vaca Key, FL	2009	Southeast	42		Vaca
Conch Key, FL	2006	Southeast	42		Conch
Palm Beach Co, FL	2006	Southeast	42		Palm

N = number of individual mosquitoes; Analyses = indicates which populations were included in SNP analyses and in one of the DIYABC analyses described in Methods. All populations were included in microsatellite genotyping and subsequent analyses of genetic diversity and population structure; Population abbreviation = abbreviations used in supplementary material: S1, S4 and S5 Figs.

For convenience, we have grouped the samples into five broad geographic regions referred to throughout this paper as Southern California, Northern California, Southwest US, South Central US, and Southeast US. The regions are described in Table 1 and shown in Fig 1B.

### DNA extraction and genotyping

Whole genomic DNA was extracted from 286 whole adult mosquitoes from ten CA sites collected in 2015 using the Qiagen DNeasy Blood and Tissue kit according to manufacturer instructions, including the optional RNAse A step. All individuals were genotyped at 12 highly variable microsatellites, as in Brown et al. [15]. The microsatellite loci are A1, B2, B3, A9 (tri-nucleotide repeats), and AC2, CT2, AG2, AC4, AC1, AC5, AG1, and AG4 (di-nucleotide repeats) [15, 24]. These loci have been validated previously for their ability to distinguish *A*. *aegypti* populations around the world [15].

A total of 107 individuals from ten CA samples (as noted in Table 1) were genotyped at 50,000 single-nucleotide polymorphisms using the high-throughput genotyping chip, Axiom_aegypti1 [25]. Cost prohibited the genotyping of all individuals, so we chose 5 Northern and 5 Southern CA populations instead. After excluding individuals that did not genotype at all microsatellites (likely due to poor DNA quality), we chose 6–12 arbitrary individuals from each population. These data were pruned as described below. Genotyping was subsequently conducted by the Functional Genomics Core at University of North Carolina, Chapel Hill.

All SNP data is available in S1 File as a VCF file, and all microsatellite data is available in S2 File. Additionally these data will be publicly available at Vectorbase.org, Population Biology Project ID: VBP0000177.

### Genetic diversity

All microsatellite loci were tested for within-population deviations from Hardy-Weinberg equilibrium and for linkage disequilibrium among loci pairs using the online version of GENEPOP [26, 27] with 10,000 dememorizations, 1,000 batches, and 10,000 iterations per batch for both tests. To correct for multiple testing, a Bonferroni correction was applied at the 0.05 α level of significance.

Observed heterozygosity (H_O_) and expected heterozygosity (H_E_) were calculated using the software GenAlEx 6.5 [28, 29], and allelic richness was estimated by rarefaction (N = 30) using the software HPRARE [30].

### Population structure

To identify likely genetic clusters and possible origins for each cluster, we used a Bayesian clustering method implemented by the software STRUCTURE v. 2.3.4 [31]. STRUCTURE identifies K genetic clusters and estimates what proportion of each individual’s ancestry is attributable to each cluster, with no *a priori* location information about the individuals. Twenty independent runs were conducted at K = 1–15 for the full set of CA and North American reference populations and at K = 1–12 for the subset of just CA populations. We ran each for 600,000 generations with 100,000 discarded as burn-in, assuming an admixture model and correlated allele frequencies. The optimal number of K clusters was chosen using the guidelines from Prichard et al. [31] and the Delta K method [32, 33]. The results were visualized using the program DISTRUCT v.1.1 [34].

To further explore population structure, discriminant analyses of principle components (DAPC), Principle Component Analyses (PCA), and plots illustrating F_ST_ values were created using the Adegenet package v. 2.0.2. [35], available on R software v. 3.2.4 and RStudio v.0.99.893 [36]. DAPC optimizes variation between clusters while minimizing variation within them. Data are transformed using a PCA and then clusters are identified using discriminant analysis. We assessed genetic differentiation among population pairs by calculating F_ST_ values with GenoDive v. 2.0b27 [37]. We also ran isolation by distance (IBD) analyses for all populations and for all CA populations using Genodive v. 2.0b27.

While the SNP chip has 50,000 probes, only 27,674 passed the initial stringent testing requiring unambiguous genotyping, biallelic and polymorphic markers, and Mendelian inheritance [25]. Further filtering was done using PLINK v.1.9 to exclude alleles showing up in <1% of samples as these could be genotyping errors, as well as loci not conforming to Hardy-Weinberg expectations (threshold of 0.00001), and those that genotyped in <98% of the samples [38, 39]. These filtering parameters are standard for SNP chip data [40–43]. The dataset contained 15,698 SNPs after this filtering.

For SNP data, we ran four runs with the Bayesian program fastSTRUCTURE to estimate the number of genetic clusters and calculate ancestry fractions for each individual given K numbers of genetic clusters [44]. The results were visualized using DISTRUCT v.1.1. For comparison, we also used the maximum likelihood software Admixture 1.3.0 and the CV error method described in the software’s manual to estimate the number of genetic structures and visualize the ancestry fractions calculated for each individual [45]. PCA analyses were conducted in both Adegenet and PLINK and plotted in R v. 3.2.4.

### Inferring colonization history

**Fig 2:**
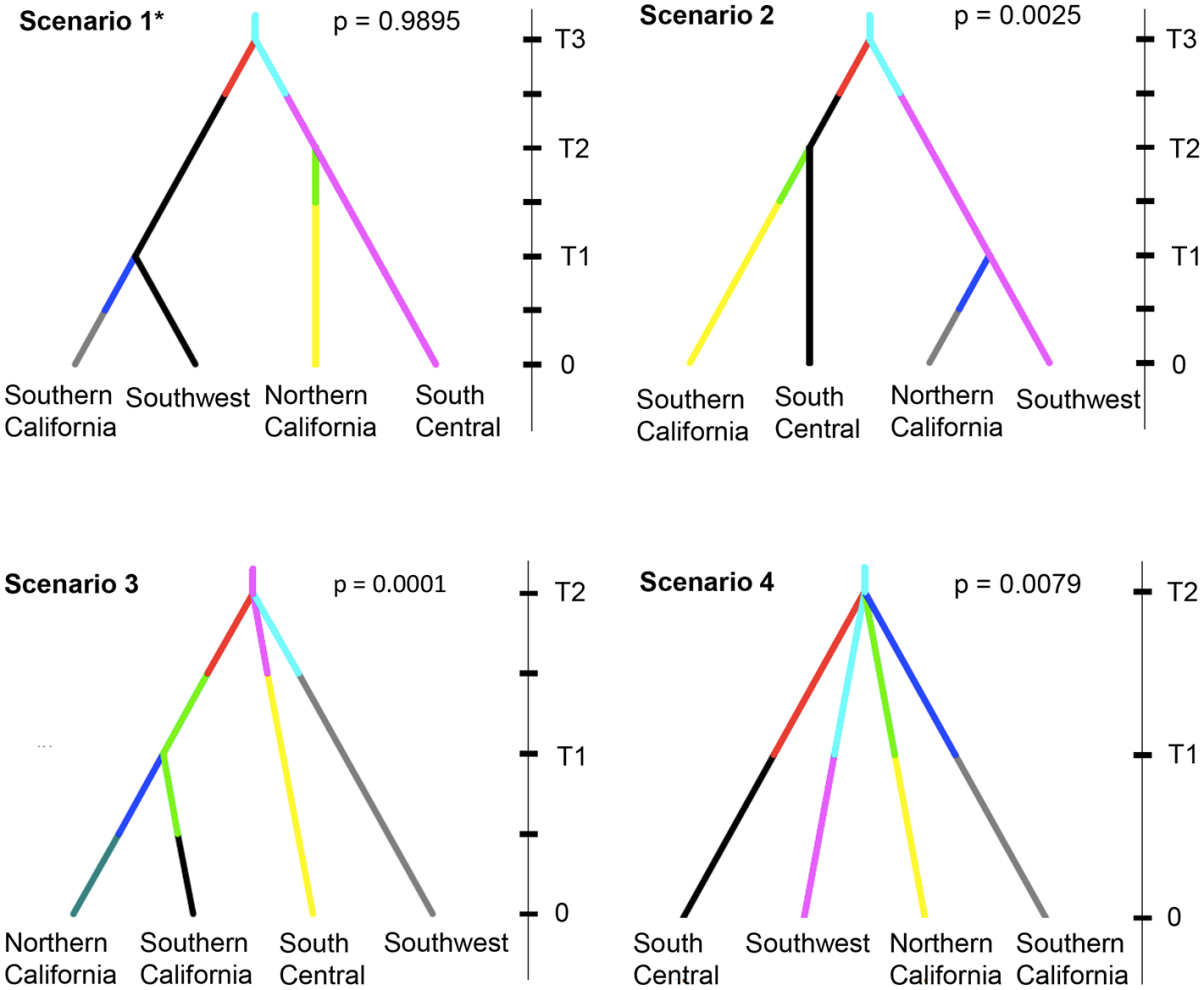
Colonization scenarios tested by DIYABC. Time is indicated on the y-axis. Time 0 corresponds to the present and time increases towards the past. The four figures show four different colonization scenarios, as described in the methods with their corresponding support values (“p” indicates the posterior probability of each scenario). Lineages are drawn in different colors. Changes in line color suggest possible changes in effective population size through time.

**Fig 3:**
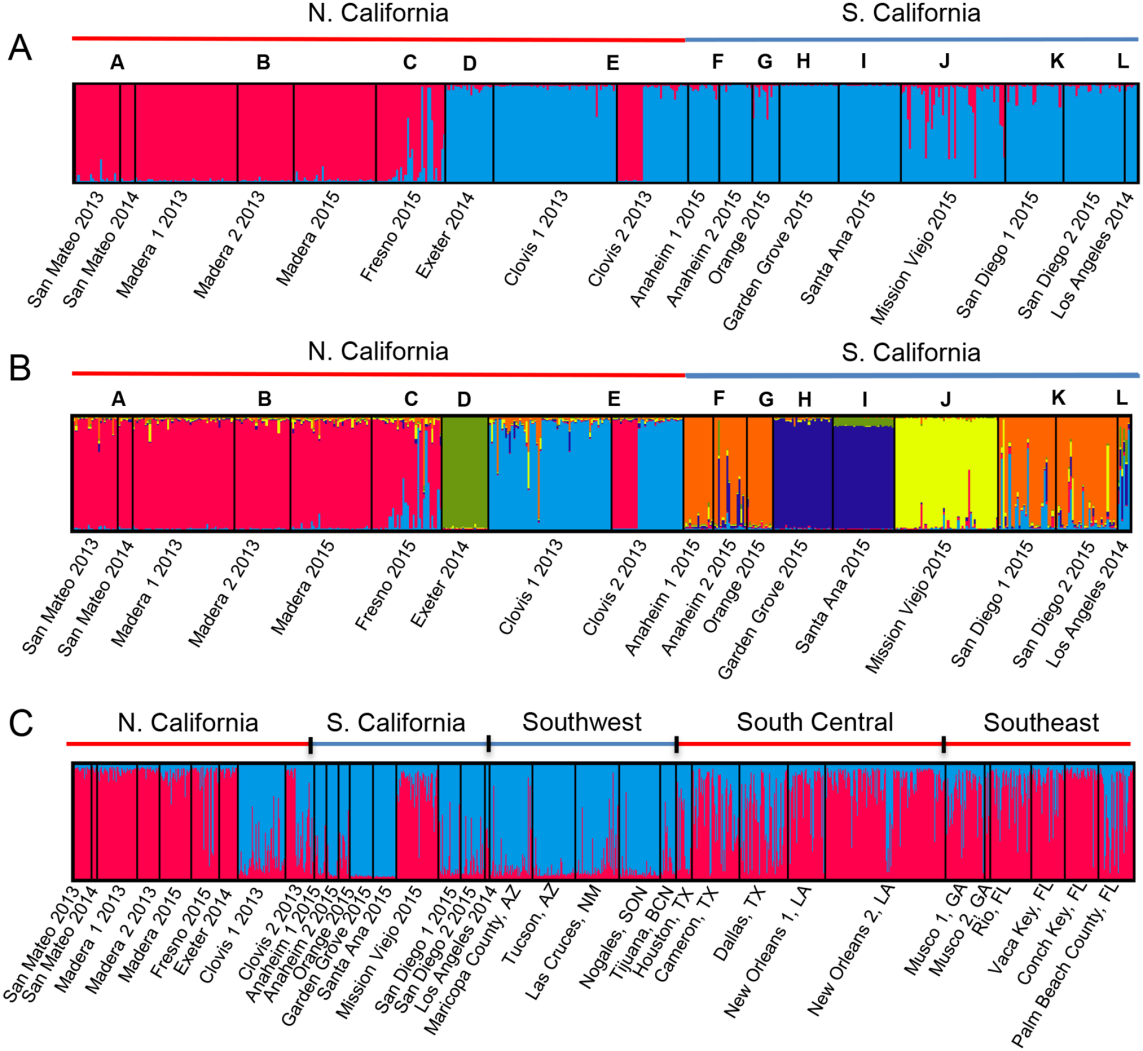
Genetic structure of California and North American populations using microsatellite loci. In these plots, each vertical bar represents an individual. The proportion of each color assigned to an individual represents the proportion of the individual’s ancestry attributable to each of K theoretical genetic clusters. Letters within the plot refer to city as in Fig 1 and Table 1. (A) California populations (K = 2). (B) California populations (K = 8). (C) California and North American populations (K = 2).

We inferred demographic history and estimated relevant parameters using microsatellite data and Approximate Bayesian Computation methods [46] as implemented by the program DIYABC [47]. Four colonization scenarios were tested to determine if the current Californian populations are more likely to be the result of one or two introduction events (Fig 2). In the first scenario, Northern California populations originate from an invasion from South Central US, and Southern California populations from an invasion from the Southwest US. In the second scenario the origins are reversed; Northern California comes from Southwest and Southern California from South Central. The third scenario depicts just one invasion into California, and the fourth scenario is a neutral model in which all four populations branch from a common ancestor at the same time. We ran the analysis using two different datasets. First, to reduce the excess noise that can result from grouping disparate populations together, we ran the analyses with regional groups that were made up of 133 randomly chosen individuals from populations that were representative of the geographic regions, as identified in STRUCTURE (Fig 3C) and noted S1 Table. For example, San Mateo, Madera, and Fresno always clustered together, so they were chosen to represent Northern California (Fig 3). To assess whether we were biasing the analysis by excluding some populations in this first test, we ran the analysis again this time forming the four regional groups from 217 randomly selected individuals from all populations from the respective geographic region. In both cases, the number of individuals was chosen based on the number of individuals in the smallest of the four groups.

For the DIYABC analyses, we first simulated 1,000,000 datasets for each scenario, resulting in 4,000,000 total simulated datasets. To determine which scenario was most supported by the data, we evaluated the relative posterior probability using a logistic regression on the 4,000 (1%) simulated datasets closest to the observed dataset. To estimate demographic parameters, we chose scenario 1 and estimated posterior distributions of parameters taking the 1,000 (1%) closest simulated datasets, after applying a logit transformation of parameter values. To evaluate confidence in the posterior probability of scenarios (in the form of Type I and Type II errors), we used a logistic regression on 250 test datasets simulated for each scenario with the same values that produced the original dataset. Priors and parameters are provided in S3 and S4 Tables.

## Results

### Genetic diversity

For microsatellites, 17 out of 2,241 (0.76%) loci pairs were found to be in linkage disequilibrium, and 3 out of 360 (0.83%) locus-population pairs were not in agreement with Hardy Weinberg equilibrium after Bonferroni correction for multiple comparisons. This is consistent with previous work indicating that these 12 microsatellites are single-copy and can be treated as independent loci for population genetic analyses [15, 16].

Genetic diversity was significantly lower within CA populations than among other North American populations (Table 2 and S1 Table). The mean observed heterozygosity +/- the standard deviation (SD) for CA sites was 0.45±0.088, and the mean of all other sites included in analysis was 0.57±0060 (Student’s t-Test, p = 0.0079). This trend was largely due to Southern California populations; 5 out of the 10 populations with the lowest heterozygosity in this analysis were located in Southern California. Similarly, the mean estimated allelic richness for California, 3.05±0.64, was lower than the mean of other populations, 3.95±0.51 (Student’s t-Test, p = 0.0013), due to Southern California. The mean allelic richness of the Southern California populations (2.62±0.41) was significantly lower than the mean from the Northern California populations (3.48±0.52) (Student’s t-Test, p<0.0001). Nine out of 10 of the analyzed sites with the lowest allelic richness were from California, and 8 of these were from Southern California.

**Table 2:** Microsatellite genetic diversity by region.

Region	H_o_±SD	H_e_±SD	AR (N = 30) ±SD
Northern California	0.53±0.066	0.52±0.026	3.48±0.52
Southern California	0.46±0.097	0.42±0.074	2.62±0.41
South Central US	0.53±0.062	0.54±0.083	3.89±0.70
Southwest US/MX	0.57±0.038	0.55±0.016	3.59±0.34
Southeast US	0.61±0.056	0.61±0.11	4.19±0.32

H_o_ = observed heterozygosity; H_e_ = expected heterozygosity; AR = allelic richness estimated by rarefaction (N = 30 genes)

### Population structure

Among California populations, pairwise F_ST_ values ranged from 0.0010 to 0.42 (S2 Table). Excluding Exeter as an outlier, pairs of Northern California sites had low mean F_ST_ values (0.060±0.051 SD). In contrast, Exeter and the Southern California sites had significantly higher mean F_ST_ values when paired with themselves (0.26±0.11 SD) or with all the CA populations (0.26±0.10) (Student’s t-Test, p<0.0001). S1 Fig illustrates this pattern graphically and in the context of all analyzed North American populations. The IBD analyses on all the populations did not show a correlation between distance and F_ST_ values (Spearman’s r = -0.19; p = 0.010; R^2^ = 0.036), and the IBD test on just the populations from CA was not significant (Spearman’s r = 0.11; p = 0.14; R^2^ = 0.011).

In regard to overwintering, the F_ST_ values between Madera 2015 and the two Madera 2013 populations is lower (0.01 and 0.047) than between Madera 2015 and any of the sites in the Central South populations (range = 0.091–0.15). The F_ST_ between San Mateo 2014 and San Mateo 2015 is higher (0.16) than between San Mateo 2014 and some of the Central South populations, such as New Orleans (0.13).

Bayesian clustering analysis for microsatellite data on all CA populations identified two primary clusters (K = 2), which divided the Northern California populations from the Southern California populations with the exception of Exeter and most of the Clovis individuals (Fig 3A). At higher K values, STRUCTURE showed a high level of population differentiation between each of the southern California populations, Exeter, and the two Clovis populations (Fig 3B and S2 Fig). In contrast, San Mateo, Madera, and Fresno populations always clustered together (Fig 3B and S2 Fig). At higher Ks, Bayesian clustering analysis that included Northern CA, the South Central, and the Southeast consistently showed that San Mateo 2013, San Mateo 2014, Madera 2013, and Madera 2014 formed a cluster that was separate from the South Central populations (eg. S3 Fig). The results from these higher K values were also found with the SNP data, as described below.

A PCA analysis using microsatellites from California populations found that the first Principal Component accounted for 13.17% of the variation and the second accounted for 9.71%. Using the same data for a DAPC analysis, the “find.clusters” command in Adegenet found the data could best be described by 12 clusters (S4 Fig). The first axis on the DAPC plot corresponds with the north-south gradient and explains 27.26% of the total variance; the second axis highlights the uniqueness of Exeter and explained 20.21% of the total variance (S4 Fig). In a DAPC plot using populations as priors, the first axis corresponds relatively well to the north-south gradient and explained 38.67% of the total variance, while the second axis explained 17.70% of the total variance (S5 Fig).

**Fig 4:**
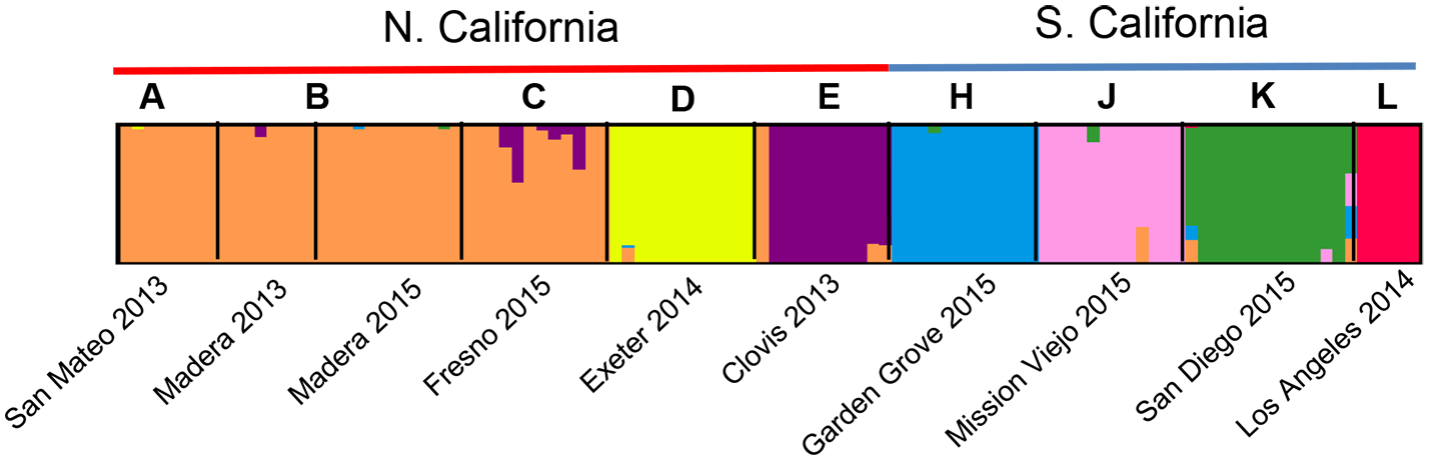
Genetic structure within California and North American populations using SNP data. The fraction of each vertical bar assigned to each color represents the proportion of that individual’s ancestry attributable to each of 6 K theoretical genetic clusters.

**Fig 5:**
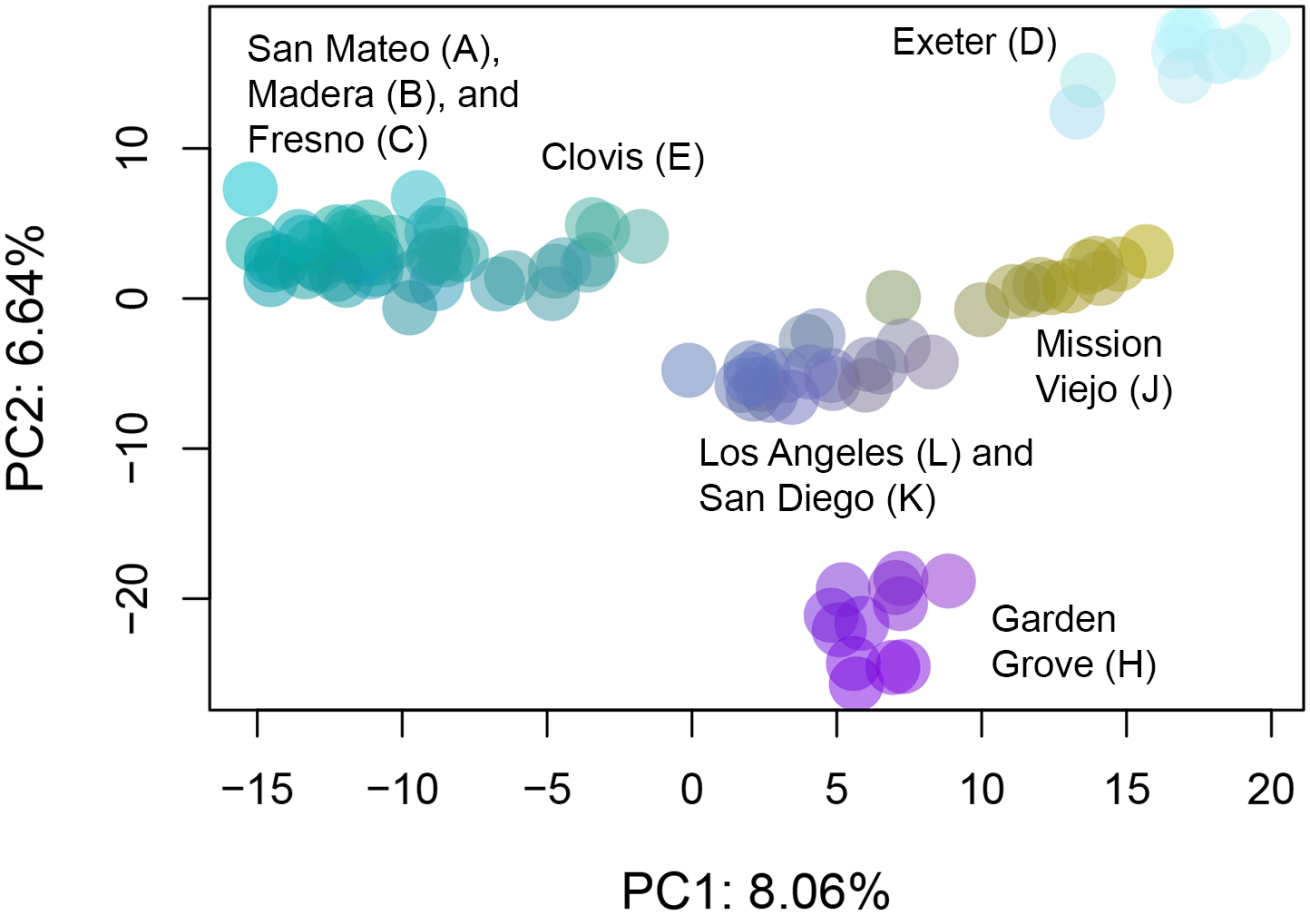
Population structure of California populations using SNP data and Principle Component Analysis. Data were transformed using PCA and plotted as a function of the first two principle components.

Analysis of the SNP data largely reinforced the microsatellite results. Seven genetic clusters were identified by fastSTRUCTURE and six by Admixture’s CV Error method of K selection on the final dataset of 15,698 SNPs (Fig 4 and S6 Fig). San Mateo, Madera, and Fresno clustered together, and each of the other populations formed its own genetic cluster (although Admixture did not distinguish between San Diego and Los Angeles) (Fig 4 and S6 Fig). The clusters observed in the PCA on the SNP data corresponded to those identified by Admixture (Fig 5). Using Adegenet, the first Principal Component explained 8.06% of the variation and was correlated with the north to south gradient, with the exception of Exeter (Fig 5). The second Principal Component accounted for 6.64% of the variation and highlighted the uniqueness of Garden Grove and Exeter.

Including microsatellite data from all analyzed populations from this study (Table 1), the Bayesian clustering method implemented by STRUCTURE identified two ancestral groups (K = 2) (Fig 3C). Most of the Northern California populations clustered with the South Central US and Southeast US populations, while most of the southern California populations clustered with the Southwest US populations (Fig 3C).

### Invasion events

The Bayesian clustering method implemented by STRUCTURE showed Northern California clustering with the South Central and Southeast regions, and Southern California clustering with the Southwest region. Since Gloria-Soria et al. showed that Houston or New Orleans was the likely origin of the Northern Californian populations [20], we used a simulation to test the hypothesis that Northern California populations originated from South Central populations and that Southern California populations originated from Southwest populations. We ran the analysis first with individuals from populations that were representative of their regions (Table 1), and secondly without excluding any populations. Since the results are similar (S3 and S4 Tables) with one described exception, all results refer to the first analysis unless otherwise noted.

Evaluation of the relative posterior probability of each of the four competing scenarios in Fig 2 supported Scenario 1 as the most plausible invasion scenario (S3 Table), in which Northern California populations split from the Central South, and Southern California populations split from the Southwest populations (p = 0.990 CI_95%_: 0.983–0.996). Scenario 4, the neutral model in which the four populations diverge from the same ancestor simultaneously, had the next highest posterior probability (0.0079 CI_95%_: 0.0019–0.0138).

The type I error rate was 0.14, and the type II error rates under the three other scenarios were 0.16, 0.11, and 0.064. The estimated time of divergence of the Central South from the Southwest populations was 4,200 generations (approximately 420 years), and the 95% credible interval was 1,730–5,900 generations assuming 10 generations/year. The estimated time of divergence of Northern California populations from South Central populations was 292 generations or approximately 29 years (95% credible interval = 61–853 generations), and the estimated time of divergence of Southern California from the Southwest was 224 generations or approximately 22 years (95% credible interval = 39.9–774). Full details are provided in S3 Table.

The results from the second analysis (in which no populations were excluded) were mostly similar including a high support for Scenario 1 (p = 0.9995 CI_95%_: 0.9991–0.9999). The estimated time of divergence between Southern California and Southwest was approximately 35 years (mean = 348, 95% credible interval = 66.9–893), and the estimated time of divergence between Northern California and the South Central/Southeast was approximately 8 years (mean = 78.0, credible interval = 15.6–414). Full details are provided in S4 Table.

## Discussion

Our analyses suggest multiple introductions of *A*. *aegypti* into CA that came from at least two different regions in North America. As previously shown, the populations in Northern California likely originated from mosquitoes that were introduced from the South Central region of the US [20]. The results of this study also suggest that a second introduction event likely occurred from the Southwest/northern MX region, and that these mosquitoes gave rise to the current populations found in Southern California. Given the considerable distance between the Northern CA and Southern CA populations (>275km from Exeter [E] to Los Angeles [F]) and the recentness of the invasions, it is not surprising that the two groups maintain distinct signatures of their genetic ancestry. As more populations are discovered between Northern and Southern CA (for example, Kern County), it would be interesting to add them to this analysis to identify whether the north-south break is clean or if the transition occurs as a cline. Identifying the area where the two clusters meet could eventually help us understand the factors leading to this genetic break.

The DIYABC analysis using representative populations suggests that the Northern California lineage diverged from the South Central lineage 292 generations ago, estimated to be ~29 years, and that the Southern California lineage diverged from the Southwest/MX lineage ~22 years ago. These estimates have large credible intervals, so we cannot tell which invasion into California happened first. The lower bounds of the intervals containing 95% probability are both more than 3 years, so it seems likely the invasions occurred at least a year prior to the initial detection of *A*. *aegypti* in 2013. In our second run of DIYABC, which did not exclude individuals from potential outliers (Clovis and Exeter), a more recent time of divergence (~8yrs) was estimated between Northern California and South Central/Southeast. This is more consistent with personal communications from CA vector control professionals who think it is unlikely the invasion could have occurred more than a year or two prior to initial detection.

Invasion events are often accompanied by a bottleneck in population size and subsequent decrease in genetic diversity, especially allelic richness. Consistent with Gloria-Soria et al. [20], we found that Northern California populations have similar levels of genetic diversity and allelic richness as other US populations. However, Southern California populations are less genetically diverse. This relatively low diversity is a possible signature of bottleneck(s) caused either by relatively recent founder effects and/or vector control measures that reduced *A*. *aegypti* population size.

We observed significant population structure with genetic differentiation even among populations in close geographic proximity, particularly among Southern California populations. For example, Anaheim, Orange, Garden Grove, and Santa Ana are each less than 12km apart from the others, but they are genetically distinguishable (S2 Fig). The dense highway system and highly discontinuous human habitats in Southern California could well cause *A*. *aegypti* to be broken into almost entirely isolated small local populations given the evidence suggesting this mosquito avoids crossing major roads/highways [48]. Similarly, Clovis and Fresno are less than 12km apart but genetically distinct (Figs 3 and 4). On the other hand, San Mateo is genetically indistinguishable from Madera and Fresno, despite the >200 km between them. Likely because of patterns like these, isolation by distance analyses did not explain the population structure in CA or throughout the regions sampled.

The large pairwise F_ST_ values between Southern California sites may indicate limited gene flow among the populations, consistent with the relatively short active dispersal distance that has been found for *A*. *aegypti* [23]. The observations are consistent with the possibility that effective *A*. *aegypti* control in one locality may not be easily influenced by migration from neighboring localities. Further analysis taking into account both timing of invasion and connectivity through human transportation routes may help us understand the patterns we see in the structure of both Southern and Northern CA populations.

In two cases, we analyzed CA populations from the same location from two different years: Madera 2013 and 2015, and San Mateo 2013 and 2014. We found strong evidence for overwintering in Madera and mixed results for San Mateo. The F_ST_ values between Madera 2013 and 2015 were lower than any of the F_ST_ values between Madera and populations from South Central US, suggesting this is a stable population and not one recolonized each year. The genetic diversity also changed very little: for example, the allelic richness was 3.5 in 2013 and 3.6 in 2015. San Mateo 2013 and San Mateo 2014 did not follow these patterns, and the small sample size of San Mateo 2014 (N = 7) could be a factor. It is unlikely that this change in genetic diversity is due to recolonization of the area by another northern California population because the mosquitoes were collected from the same confined area in 2013 and 2014, and were not detected elsewhere in the county. In both cases the populations are always part of the same genetic cluster determined with Bayesian clustering (e.g. Fig 3), consistent with a previous study demonstrating that temporal differentiation does not obscure geographic structure in CA *A*. *aegypti* populations [16]. Strikingly, STRUCTURE analyses of North American populations at higher K values showed that San Mateo and Madera formed a genetic cluster separate from other Central South and Southeast populations (S3 Fig). This temporal stability indicates that some populations in Northern California are stable and likely continuously breeding *in situ*, rather than being recolonized by their original source. In five cases, we have included multiple populations from the same city and the same years. Except for a few anomalies (e.g. a subset of Clovis), these populations did not show genetic differentiation, suggesting no population structure within a city.

Our paper is the first to combine SNP data and microsatellites to address the population structure and origins of the CA *A*. *aegypti*. We found the same genetic structure using both types of markers, which speaks to the robustness of our methods and results. Our results suggest the microsatellite markers, which are more cost-efficient, are sufficient for these types of analyses. However, we expect the SNP chip will continue to provide essential information in other situations, for example, at finer-scales, when fewer individuals are available, or when building phylogenies.

That *A*. *aegypti* invaded CA multiple times, probably years before its first detection in 2013, has important implications for vector control. It implies CA and other regions with a temperate climate may be more vulnerable to invasion than previously thought. Additionally, we find that CA *A*. *aegypti* populations near one another are often genetically distinct. A challenge to providing effective vector control is the potential for reinvasion in targeted regions. At least in Southern CA it appears that there is little evidence for extensive migration and gene flow among populations. The disparate genetic backgrounds in these *A*. *aegypti* populations may represent populations capable of responding differently to control measures such as pesticides, or perhaps these populations may have different vector competence for infectious pathogens. CA has one of the most extensive mosquito-monitoring systems in the US, so the possibility that *A*. *aegypti* was in CA years before detection may mean mosquito invasions have occurred elsewhere in the US but escaped notice. Understanding and accounting for the invasion dynamics of *A*. *aegypti* will continue to be essential for detecting new invasions, monitoring vector presence, and preventing disease outbreaks in California and other regions.

## Supporting information

S1 Fig Illustrative plot of pairwise F_ST_ values between all North American populations. Each box represents the F_ST_ value between the corresponding populations on the horizontal and vertical axes. The darker the color of the box, the higher the F_ST_ value is. The legend on the bottom shows how each color corresponds to F_ST_ values between 0.05 and 0.3.
(TIF)

S2 Fig Genetic structure within Northern and Southern California using microsatellite data. Each vertical bar represents an individual. The proportion of each color assigned to each individual represents the proportion of that individual’s ancestry attributable to each of K theoretical genetic clusters. Letters within the plot refer to city as in Fig 1 and Table 1. (A) Northern California populations (K = 8). (B) Southern California populations (K = 8).
(TIF)

S3 Fig Genetic structure within North America using microsatellite data. Each vertical bar represents an individual. The proportion of each color assigned to each individual represents the proportion of that individual’s ancestry attributable to each of K theoretical genetic clusters. (A) K = 3. (B) K = 4.
(TIF)

S4 Fig Population structure of California populations using DAPC (microsatellite data). A) This chart shows the composition of each of the 12 inferred genetic clusters; the larger the black box, the more individuals included. For example, inferred group 4 contains all the individuals from Mission Viejo and inferred group 10 contains a mixture of the individuals from the two San Diego populations. (See Table 1 for population codes.) B) A plot of the inferred genetic clusters using the first two principle components as axes.
(TIF)

S5 Fig Population structure of California populations using DAPC and populations as priors (microsatellite data). In this plot, populations were selected *a priori* based on regional location and the first two principle components served as the axes.
(TIF)

S6 Fig Genetic structure within California and North American populations using SNP data. The fraction of each vertical bar assigned to each color represents the proportion of that individual’s ancestry attributable to each of 6 theoretical genetic clusters. Letters within the plot refer to city as in Fig 1 and Table 1.
(TIF)

S1 File VCF file of all CA SNP data. SNP data from the CA populations indicated in Table 1 (not pruned). Reference genome used is AaegL1.
(VCF)

S2 File Excel file of all microsatellite data used in alayses. Microsatellite data from all populations indicated in Table 1. Missing data is indicated with -9.
(XLSX)

S1 Table Genetic diversity by population. H_o_ = observed heterozygosity; H_e_ = expected heterozygosity; AR = allelic richness estimated by rarefaction (n = 30 genes)
(DOCX)

S2 Table F_ST_ values between all populations included in analyses. (XLSX)

S3 Table DIYABC analysis: Introductions into California. Regional groups composed of individuals from representative populations from each region.
(DOCX)

S4 Table DIYABC analysis: Introductions into California. Regional groups composed of individuals from all populations from each region.
(DOCX)
