# Supplementary figures and images for: Multiple introductions of the dengue vector, Aedes aegypti, into California
Source: PLoS Negl Trop Dis. 2017 Aug 10;11(8):e0005718. doi: 10.1371/journal.pntd.0005718 (PMC5552028; doi:10.1371/journal.pntd.0005718)

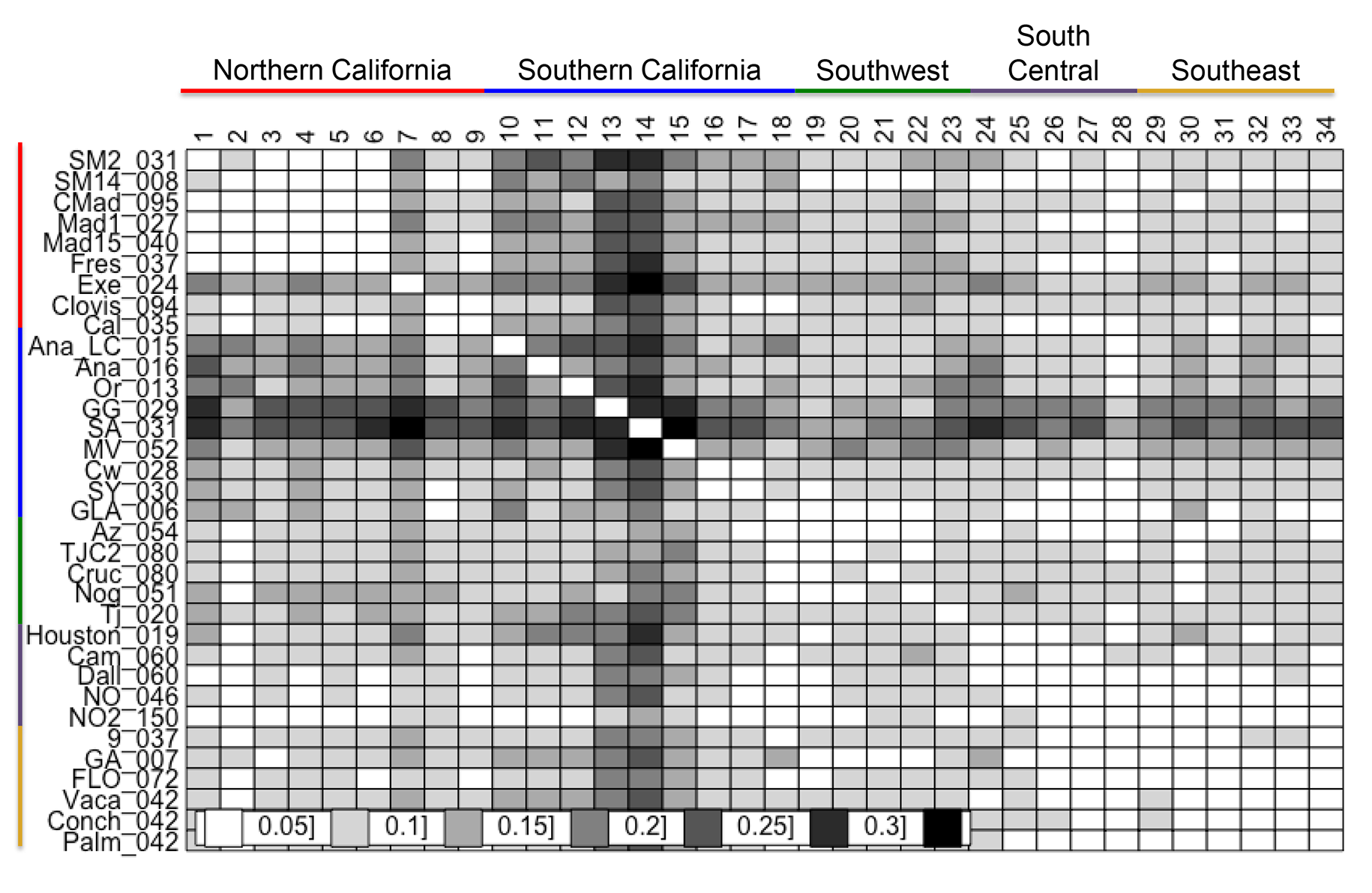

Supplement: S1 Fig — Each box represents the FST value between the corresponding populations on the horizontal and vertical axes. The darker the color of the box, the higher the FST value is. The legend on the bottom shows how each color corresponds to FST values between 0.05 and 0.3. (TIF) [file pntd.0005718.s001.tif]

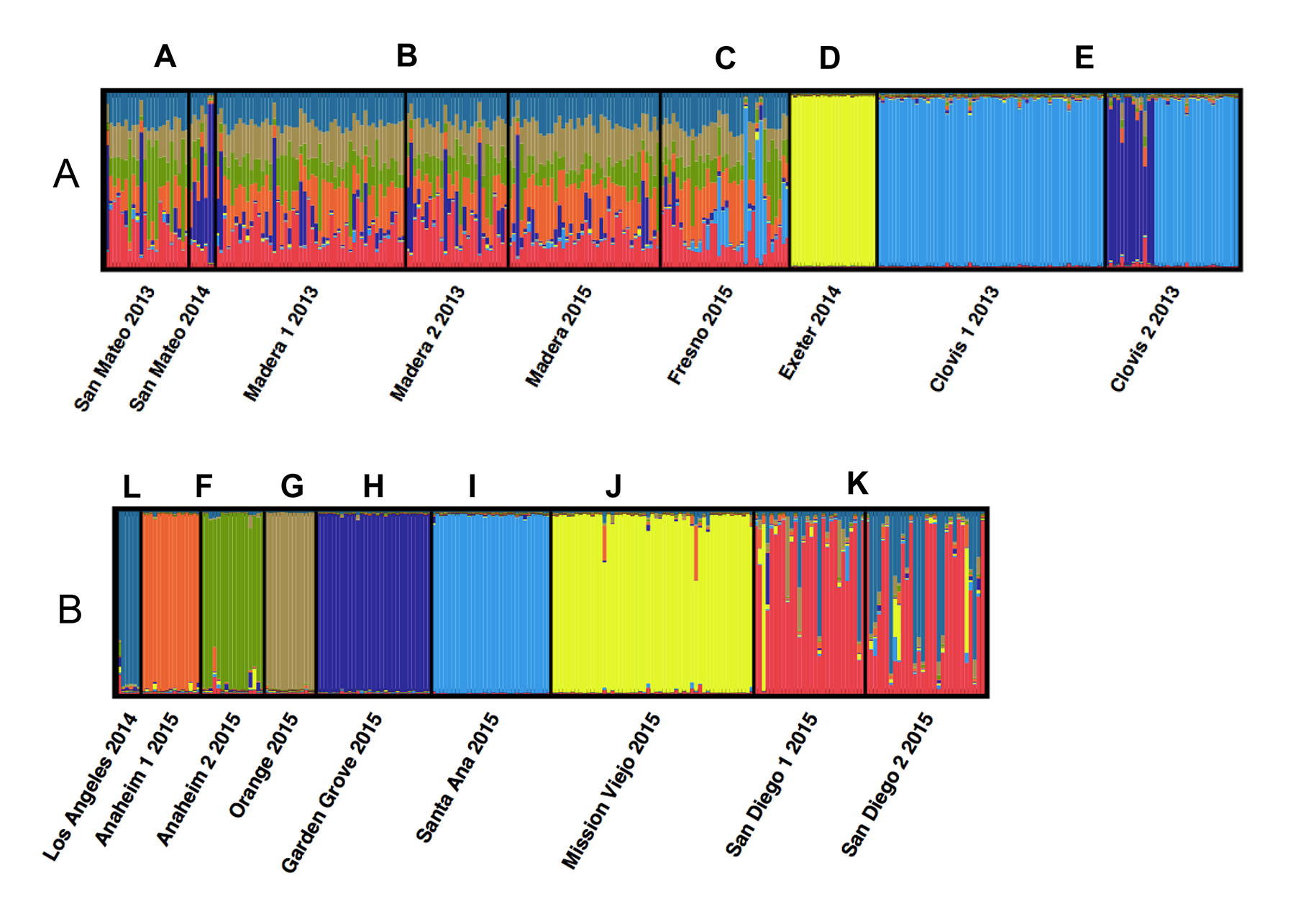

Supplement: S2 Fig — Each vertical bar represents an individual. The proportion of each color assigned to each individual represents the proportion of that individual’s ancestry attributable to each of K theoretical genetic clusters. Letters within the plot refer to city as in Fig 1 and Table 1. (A) Northern California populations (K = 8). (B) Southern California populations (K = 8). (TIF) [file pntd.0005718.s002.tif]

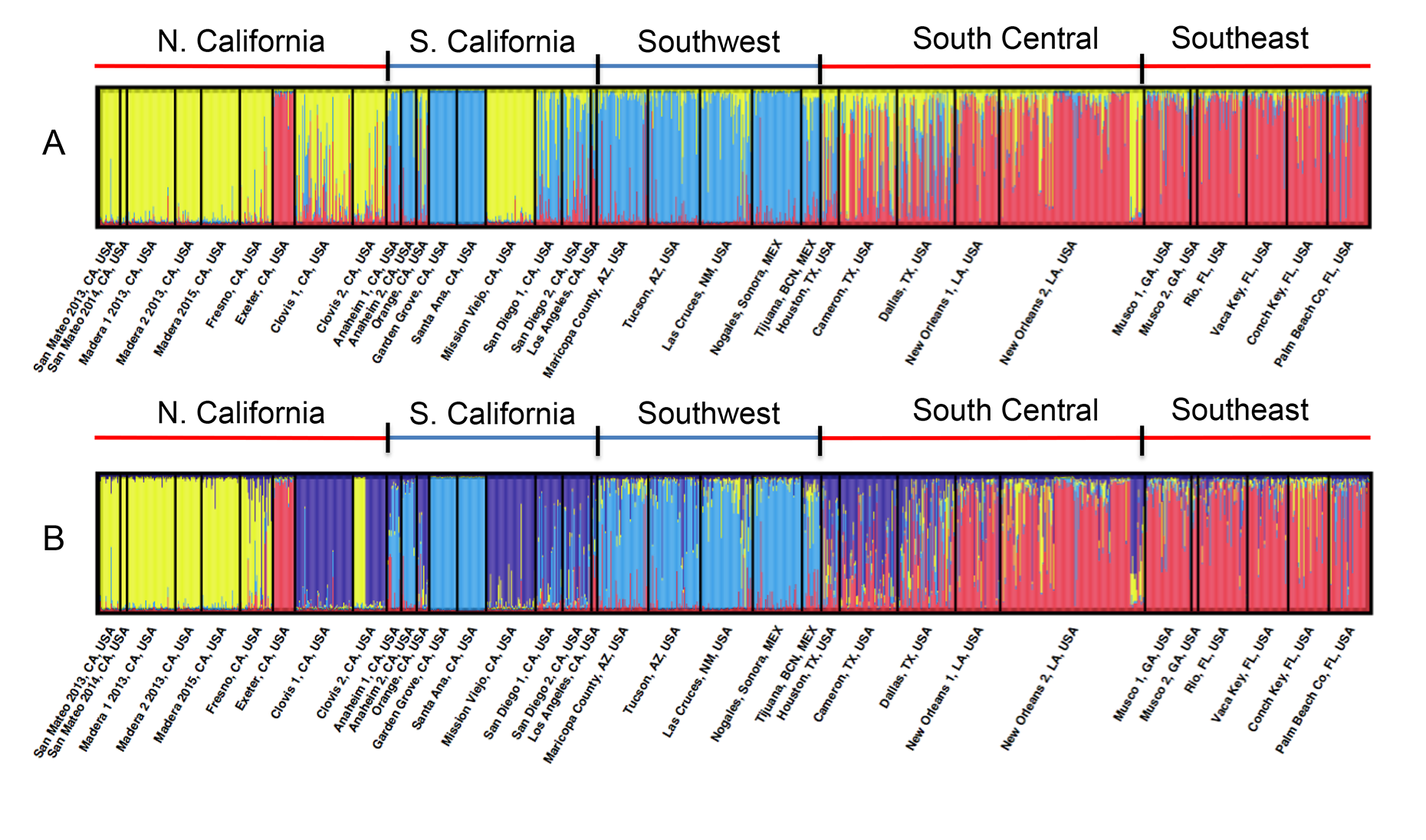

Supplement: S3 Fig — Each vertical bar represents an individual. The proportion of each color assigned to each individual represents the proportion of that individual’s ancestry attributable to each of K theoretical genetic clusters. (A) K = 3. (B) K = 4. (TIF) [file pntd.0005718.s003.tif]

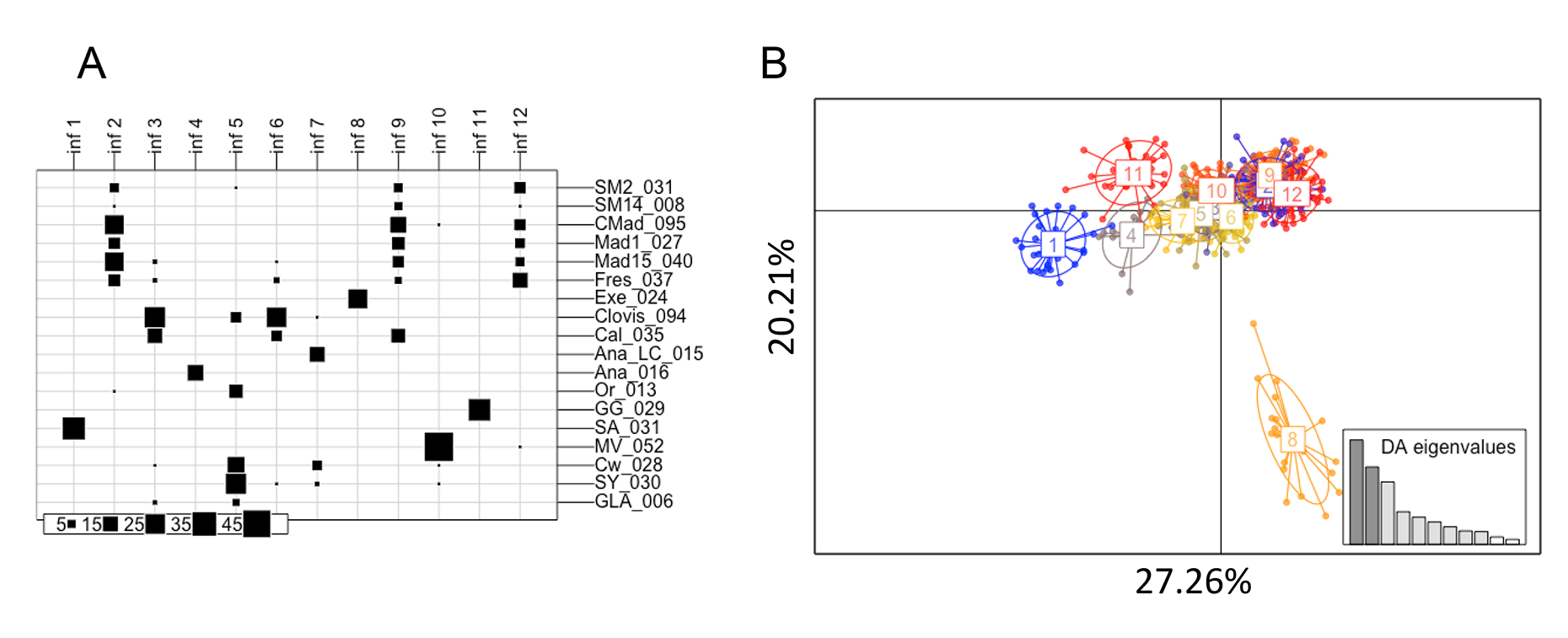

Supplement: S4 Fig — A) This chart shows the composition of each of the 12 inferred genetic clusters; the larger the black box, the more individuals included. For example, inferred group 4 contains all the individuals from Mission Viejo and inferred group 10 contains a mixture of the individuals from the two San Diego populations. (See Table 1 for population codes.) B) A plot of the inferred genetic clusters using the first two principle components as axes. (TIF) [file pntd.0005718.s004.tif]

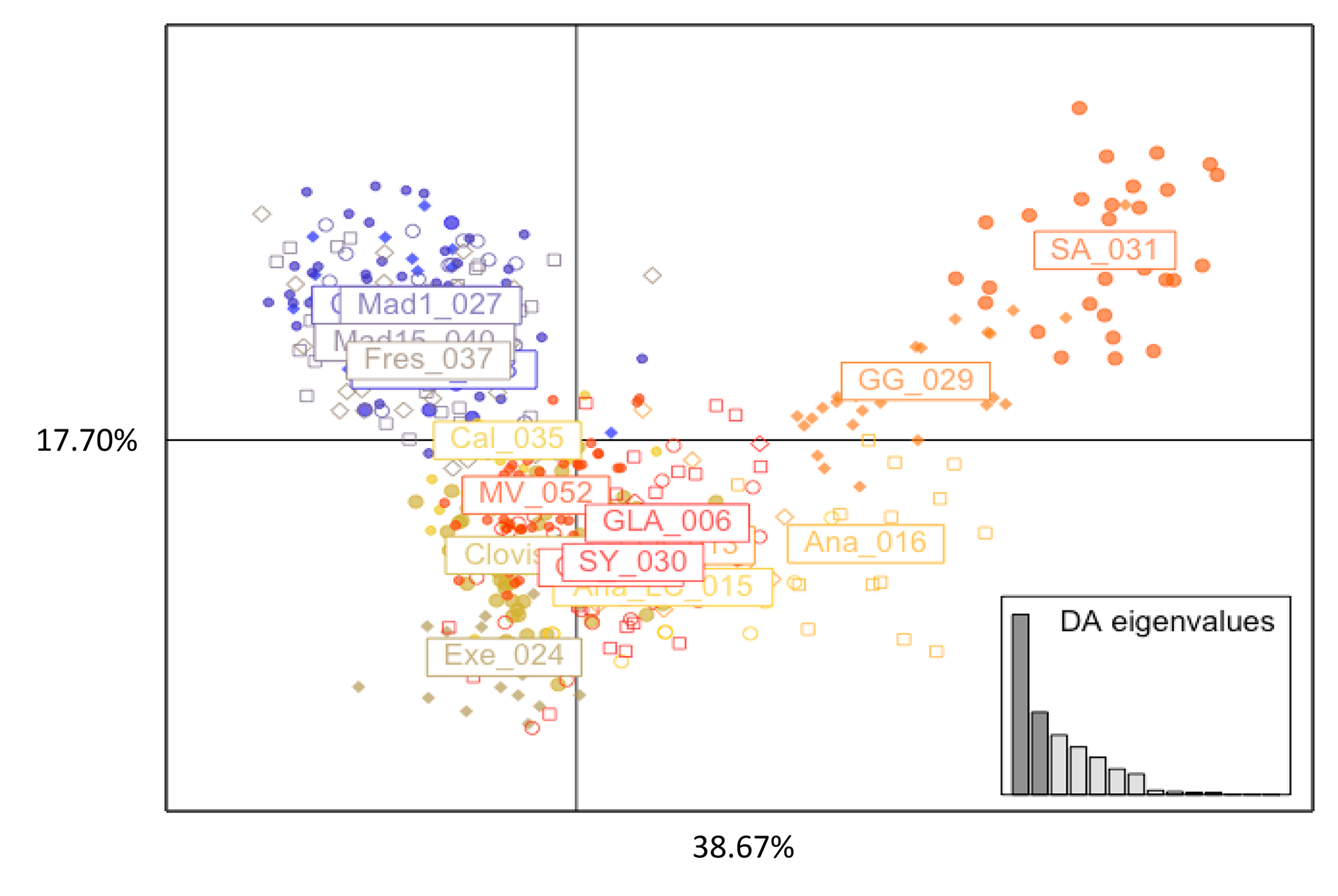

Supplement: S5 Fig — In this plot, populations were selected a priori based on regional location and the first two principle components served as the axes. (TIF) [file pntd.0005718.s005.tif]

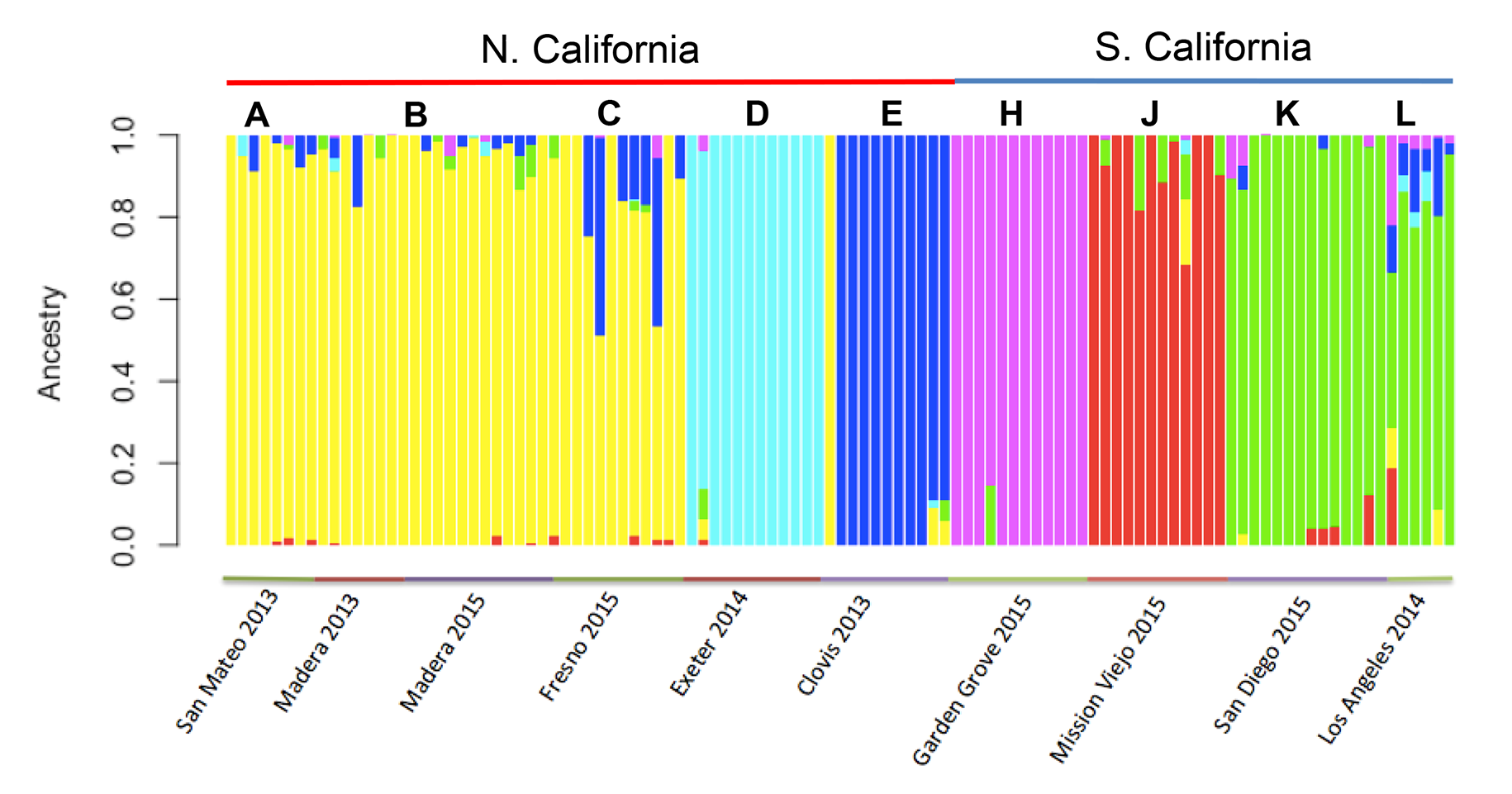

Supplement: S6 Fig — The fraction of each vertical bar assigned to each color represents the proportion of that individual’s ancestry attributable to each of 6 theoretical genetic clusters. Letters within the plot refer to city as in Fig 1 and Table 1. (TIF) [file pntd.0005718.s006.tif]
