# Supplementary material for: Multiple introductions of the dengue vector, Aedes aegypti, into California
Source: PLoS Negl Trop Dis. 2017 Aug 10;11(8):e0005718. doi: 10.1371/journal.pntd.0005718 (PMC5552028; doi:10.1371/journal.pntd.0005718)
Supplement: S1 Table — Ho = observed heterozygosity; He = expected heterozygosity; AR = allelic richness estimated by rarefaction (n = 30 genes) (DOCX) [file pntd.0005718.s009.docx]

**Table S1.** Genetic diversity by population

| **Locality** | **Year** | **Region** | **Ho** | **He** | **AR (n=30)** |
| --- | --- | --- | --- | --- | --- |
| San Mateo, CA | 2013 | Northern California | 0.496 | 0.509 | 3.71 |
| San Mateo, CA | 2014 | Northern California | 0.645 | 0.495 | 4 |
| Madera, CA (1) | 2013 | Northern California | 0.533 | 0.541 | 3.56 |
| Madera, CA (2) | 2013 | Northern California | 0.605 | 0.533 | 3.32 |
| Madera, CA | 2015 | Northern California | 0.506 | 0.530 | 3.09 |
| Fresno, CA | 2015 | Northern California | 0.455 | 0.550 | 4.14 |
| Exeter, CA | 2014 | Northern California | 0.514 | 0.482 | 2.45 |
| Clovis, CA (1) | 2013 | Northern California | 0.451 | 0.489 | 3.25 |
| Clovis, CA (2) | 2013 | Northern California | 0.576 | 0.548 | 3.79 |
| Anaheim, CA (1) | 2015 | Southern California | 0.578 | 0.400 | 2.18 |
| Anaheim, CA (2) | 2015 | Southern California | 0.524 | 0.478 | 2.9 |
| Orange, CA | 2015 | Southern California | 0.474 | 0.414 | 2.36 |
| Garden Grove, CA | 2015 | Southern California | 0.345 | 0.331 | 2.12 |
| Santa Ana, CA | 2015 | Southern California | 0.340 | 0.335 | 2.27 |
| Mission Viejo, CA | 2015 | Southern California | 0.336 | 0.331 | 2.62 |
| San Diego, CA (1) | 2015 | Southern California | 0.494 | 0.477 | 2.98 |
| San Diego, CA (2) | 2015 | Southern California | 0.518 | 0.505 | 3.32 |
| Los Angeles, CA | 2014 | Southern California | 0.556 | 0.497 | 2.82 |
| Maricopa County, AZ | 2013 | Southwest | 0.554 | 0.557 | 3.86 |
| Tucson, AZ | 2012 | Southwest | 0.599 | 0.565 | 3.66 |
| Las Cruces, NM | 2015 | Southwest | 0.534 | 0.523 | 3.69 |
| Nogales, SON, MEX | 2013 | Southwest | 0.551 | 0.558 | 3.75 |
| Tijuana, BC, MEX | 2013 | Southwest | 0.625 | 0.550 | 2.99 |
| Houston, TX | 2011 | South Central | 0.439 | 0.416 | 2.98 |
| Cameron County, TX | 2015 | South Central | 0.489 | 0.489 | 3.73 |
| Dallas County, TX | 2015 | South Central | 0.551 | 0.562 | 4.28 |
| New Orleans, LA | 2011 | South Central | 0.570 | 0.617 | 4.58 |
| New Orleans, LA | 2012 | South Central | 0.589 | 0.597 | 4.58 |
| Musco, GA | 2011 | Southeast | 0.641 | 0.634 | 4.45 |
| Musco, GA | 2012 | Southeast | 0.679 | 0.568 | 3.55 |
| Rio, FL | 2014 | Southeast | 0.609 | 0.627 | 4.39 |
| Vaca Key, FL | 2009 | Southeast | 0.512 | 0.589 | 4.26 |
| Conch Key, FL | 2006 | Southeast | 0.599 | 0.604 | 4.27 |
| Palm Beach Co, FL | 2006 | Southeast | 0.595 | 0.618 | 4.22 |

H_o_=observed heterozygosity; H_e_=expected heterozygosity; AR=allelic richness estimated by rarefaction (n=30 genes)
