# Supplementary material for: Multiple introductions of the dengue vector, Aedes aegypti, into California
Source: PLoS Negl Trop Dis. 2017 Aug 10;11(8):e0005718. doi: 10.1371/journal.pntd.0005718 (PMC5552028; doi:10.1371/journal.pntd.0005718)
Supplement: S3 Table — Regional groups composed of individuals from representative populations from each region. (DOCX) [file pntd.0005718.s011.docx]

**Table S3.** DIYABC analysis: introductions into California

| **Parameter** | **Details*** | **Prior** | **Posterior** |
| --- | --- | --- | --- |
| Colonization scenario | Scenario 1 – Central South is origin of Northern California, and Southwest is origin of Southern California | 0.25 | 0.9895 [0.9830, 0.9961] |
|  | Scenario 2 – Central South is origin of Southern California, and Southwest is origin of Northern California | 0.25 | 0.0025 [0.0012, 0.0038] |
|  | Scenario 3 – One introduction into California | 0.25 | 0.0001 [0.0001, 0.0002] |
|  | Scenario 4 – Neutral model | 0.25 | 0.0079 [0.0019, 0.0138] |
| Effective population size | 1. Central South (includes Houston TX, Cameron TX, Dallas TX, and New Orleans LA) | Uniform  100 – 500,000 | Mean: 77,700  Median: 41,600  Quantile 2.5%: 7,380  Quantile 97.5%: 376,000 |
|  | 3. Southwest (includes Nogales MX, Las Cruces NM, Tucson AZ) | Uniform  100 – 500,000 | Mean: 11,800  Median: 3,390  Quantile 2.5%: 640  Quantile 97.5%: 81,400 |
|  | 2. Northern California (includes Fresno, Madera, and San Mateo) | Uniform  100 – 500,000 | Mean: 191,000  Median: 166,000  Quantile 2.5%: 5,300  Quantile 97.5%: 475,000 |
|  | 4. Southern California (includes Anaheim, Orange, Garden Grove, Santa Ana, Mission Viejo, San Diego) | Uniform  100 – 500,000 | Mean: 241,000  Median: 237,000  Quantile 2.5%: 9,530  Quantile 97.5%: 486,000 |
| Split time in generations (10 generations/year) | T1. Southern California from Southwest | Uniform  20 – 1000  t1<t0 | Mean: 224  Median: 168  Quantile 2.5%: 39.9  Quantile 97.5%: 774 |
|  | T2. Northern California from Central South | Uniform  20 – 1000  t2<t0 | Mean: 292  Median: 238  Quantile 2.5%: 61  Quantile 97.5%: 853 |
|  | T3. Central South from Southwest | Uniform  20 – 10,000 | Mean: 4,200  Median: 4,330  Quantile 2.5%: 1,730  Quantile 97.5%: 5,900 |
| Mutation Rate | Microsatellite – General Mutation Model (GMM) | Uniform  9x10^-6^ – 1x10^-3^ | Mean: 2.34 x 10^-4^  Median: 1.94 x 10^-4^  Quantile 2.5%: 7.64 x 10^-5^  Quantile 97.5%: 6.44 x 10^-4^ |
| Confidence | Type I Error (simulated under scenario 1) | N/A | 0.14 |
|  | Type II Error (simulated under scenario 2) | N/A | 0.16 |
|  | Type II Error (simulated under scenario 3) | N/A | 0.02 |
|  | Type II Error (simulated under scenario 4) | N/A | 0.004 |

*Regional groups composed of individuals from representative populations from each region.
