# Supplementary material for: Multiple introductions of the dengue vector, Aedes aegypti, into California
Source: PLoS Negl Trop Dis. 2017 Aug 10;11(8):e0005718. doi: 10.1371/journal.pntd.0005718 (PMC5552028; doi:10.1371/journal.pntd.0005718)
Supplement: S4 Table — Regional groups composed of individuals from all populations from each region. (DOCX) [file pntd.0005718.s012.docx]

**Table S4.** DIYABC analysis: introductions into California

| **Parameter** | **Details*** | **Prior** | **Posterior** |
| --- | --- | --- | --- |
| Colonization scenario | Scenario 1 – Central South is origin of Northern California, and Southwest is origin of Southern California | 0.25 | 0.9995 [0.9991, 0.9999] |
|  | Scenario 2 – Central South is origin of Southern California, and Southwest is origin of Northern California | 0.25 | 0.0002 [0.0001, 0.0004] |
|  | Scenario 3 – One introduction into California | 0.25 | 0.0002 [0.0000, 0.0004] |
|  | Scenario 4 – Neutral model | 0.25 | 0.0001 [0.0000, 0.0002] |
| Effective population size | 1. Central South and Southeast | Uniform  100 – 500,000 | Mean: 67,100  Median: 38,500  Quantile 2.5%: 7,010  Quantile 97.5%: 322,000 |
|  | 3. Southwest | Uniform  100 – 500,000 | Mean: 9,230  Median: 3,050  Quantile 2.5%: 677  Quantile 97.5%: 54,600 |
|  | 2. Northern California | Uniform  100 – 500,000 | Mean: 286,000  Median: 303,000  Quantile 2.5%: 17,100  Quantile 97.5%: 492,000 |
|  | 4. Southern California | Uniform  100 – 500,000 | Mean: 262,000  Median: 279,000  Quantile 2.5%: 1,000  Quantile 97.5%: 490,000 |
| Split time in generations (10 generations/year) | T1. Southern California from Southwest | Uniform  20 – 1000  t1<t0 | Mean: 348  Median: 303  Quantile 2.5%: 66.9  Quantile 97.5%: 893 |
|  | T2. Northern California from Central South | Uniform  20 – 1000  t2<t0 | Mean: 78.0  Median: 46.7  Quantile 2.5%: 15.6  Quantile 97.5%: 414 |
|  | T3. Central South from Southwest | Uniform  20 – 10,000 | Mean: 5,260  Median: 5,490  Quantile 2.5%: 3,180  Quantile 97.5%: 5,980 |
| Mutation Rate | Microsatellite – General Mutation Model (GMM) | Uniform  9x10^-6^ – 1x10^-3^ | Mean: 2.32 x 10^-4^  Median: 1.95 x 10^-4^  Quantile 2.5%: 8.12 x 10^-5^  Quantile 97.5%: 6.26 x 10^-4^ |
| Confidence | Type I Error (simulated under scenario 1) | N/A | 0.14 |
|  | Type II Error (simulated under scenario 2) | N/A | 0.07 |
|  | Type II Error (simulated under scenario 3) | N/A | 0.02 |
|  | Type II Error (simulated under scenario 4) | N/A | 0.02 |

*Regional groups composed of individuals from all populations from each region.
